# Editorial

**Published:** 1898-03

**Authors:** 


### EDITORIAL DEPARTMENT.

F. E. DANIEL, M. D., Editor.
S. E. HUDSON, M. D., Managing Editor.
A. J. SMITH. M. D., Galveston, Associate Editor.

Published Monthly at Austin, Texas, by Drs. Daniel and Hudson. Subscription price $2.00 a year in advance.

Eastern Representatives: Messrs. Monihan & Fairchild, St. Paul Building, 220 Broadway, New York City.

Official organ of the West Texas Medical Association, the Houston District Medical Association, the Austin District Medical Society, the Brazos Valley Medical Association, the Galveston County Medical Society, and several others.

### EDITORIAL.

THE LATE QUARANTINE CONVENTION.—We publish elsewhere in this issue a synopsis of the proceedings of the Quarantine Conference held in Mobile, on the 9th of February, ult., and call attention to it. It will be seen that there were two factions represented; one in favor of doing away with State quarantine, and entrusting the whole matter of quarantine to the Federal government; the other as strongly in favor of State autonomy. Two reports were submitted; a majority report embodying the wishes of the party first mentioned; the other, the minority report, favoring State control. Both of these reports were rejected, and a compromise was effected, by the adoption of the resolution published elsewhere in this issue.

If the writer, who strongly favors States' rights, had been entrusted with the wording of the resolution, he could not have drawn up a resolution that would have suited him better.

Its adoption unanimously is most significant and gratifying. It is a signal victory for States' rights; and in the face of such an emphatic expression on the subject, surely Southern congressmen will strive to carry out the wishes of their constituents by opposing the machinations of the Supervising Surgeon General of the Marine Hospital Service, and the ill-considered bills of Vest and Caffery.

The Department of Public Health is too important to be made a sub-department and tacked on to the tail of the Treasury Department. Legislation in line with the resolution unanimously adopted at the conference will, we believe, solve the perplexing problem, and give us a quarantine system efficient and harmonious, and which does not involve any surrender of States' rights nor subject the States to any humiliation. In the resolution there is outlined exactly the relation that should exist between State and National quarantine; a relation of mutual dependence and co-operation.

